# 14,15‐epoxyeicosatrienoic acid produced by cytochrome P450s enhances neurite outgrowth of PC12 and rat hippocampal neuronal cells

**DOI:** 10.1002/prp2.428

**Published:** 2018-09-17

**Authors:** Ami Oguro, Takumi Inoue, Suguru N. Kudoh, Susumu Imaoka

**Affiliations:** ^1^ Department of Biomedical Chemistry School of Science and Technology Kwansei Gakuin University Sanda Japan; ^2^ Department of Human‐System Interaction School of Science and Technology Kwansei Gakuin University Sanda Japan

**Keywords:** epoxyeicosatrienoic acid, neurite outgrowth, P450, PC12, rat hippocampal neuronal cell

## Abstract

Polyunsaturated fatty acids, such as arachidonic acid, are accumulated in brain and induce neuronal differentiation. Arachidonic acid is metabolized to epoxyeicosatrienoic acids (EETs) and hydroxyeicosatetraenoic acids (HETEs) by cytochrome P450s. In this study, we found that 14,15‐EET and 20‐HETE‐enhanced NGF‐induced rat pheochromocytoma PC12 cell neurite outgrowth even at the concentration of 100 nmol L^−1^. LC‐MS analysis revealed that 14,15‐EET was effectively produced from arachidonic acid by rat CYP2C11, 2C13, and 2C23, and these P450s were expressed in PC12 cells. An inhibitor of these P450s, ketoconazole, inhibited neurite outgrowth, whereas inhibition of soluble epoxide hydrolase, which hydrolyzes EETs to their corresponding diols enhanced neurite outgrowth. To determine the mechanism of neurite formation enhancement by arachidonic acid metabolites, we focused on transient receptor potential (TRP) channels expressed in PC12 cells. The TRPV4 inhibitor HC067047, but not the TRPV1 inhibitor capsazepine, inhibited the effects of 14,15‐EET on neurite outgrowth of PC12. Furthermore, 14,15‐EET increased the cytosolic calcium ion concentration and this increase was inhibited by HC067047. 14,15‐EET also enhanced neurite outgrowth of primary cultured neuron from rat hippocampus. This study suggests that arachidonic acid metabolites produced by P450 contribute to neurite outgrowth through calcium influx.

**Abbreviations**
EET: epoxyeicosatrienoic acid
HETE: hydroxyeicosatetraenoic acid
P450 or CYP: Cytochrome P450
sEH: soluble epoxide hydrolase
TRP: transient receptor potential

## 1 INTRODUCTION

Polyunsaturated fatty acids, such as arachidonic acid and docosahexaenoic acid (DHA), are essential for infant development, and especially effective for neuronal diseases such as Alzheimer's disease and Parkinson's disease.^1^ Supplemental unsaturated fatty acids taken directly influence the composition and function of cerebral membranes,^2^ especially arachidonic acid rapidly accumulated in the brain during development.^3^ It has also been showed that a release of arachidonic acid by activation of phospholipase A_2_ is important in neuronal outgrowth of mouse axons.^4^

Cytochrome P450s (P450s) are monooxygenase and phase I drug metabolizing enzymes. P450s also oxidize arachidonic acid to epoxyeicosatrienoic acids (EETs) and hydroxy‐eicosatetraenoic acids (HETEs).^5^ Furthermore, EETs are metabolized to dihydroxyeicosatrienoic acids (DHETs) by soluble epoxide hydrolase (sEH). There are many HETE isomers produced by P450s, including 5, 8, 9, 11, 12, 15, 16, 17, 18, 19, and 20‐HETEs.^6^ 19‐HETE is a stimulator of renal Na^+^–K^+^‐ATPase^7^ and 20‐HETE is a potent vasoconstrictor in isolated rat aortas.^8^ EETs were initially identified as endothelium‐derived hyperpolarizing factors (EDHFs)^9^ and attracted much interest. However, EETs are now suggested to be more than just vasodilators. EETs were also found to influence many biological processes, including ion channel regulation, mitogenesis, and inflammation.^10^ There are four EET isomers, 5,6‐, 8,9‐, 11,12‐, and 14,15‐EET. 11,12‐EET and 14,15‐EET were demonstrated to be powerful mitogens, using kidney‐ and endothelium‐derived culture cells.^11^ 11,12‐EET is also involved in angiogenesis under hypoxic conditions.^12^ Although specific receptors for EETs and HETEs have not been identified, EETs activate some receptors such as Ca^2+^‐activated K^+^ channels and transient receptor potential (TRP) channels.^13^

In the brain, HETEs and EETs are released from neurons, astrocytes, and cerebral blood vessels, and contribute to cerebral blood flow and angiogenesis,^14^ but their effects on neural functions are not well understood. The neurite outgrowth of neurons is very important for the function of the central nervous system. Rat phenochromocytoma PC12 cells are used as a model system for neurodegenerative diseases and as an in vitro model system to study cell differentiation. Nerve growth factor (NGF) induces neural differentiation of PC12 cells, leading to neurite outgrowth. NGF also induces expression of phospholipase A_2_ in PC12 cells.^15^ We also used primary cultured neuron isolated from rat fetal hippocampus. In this study, we investigate biological functions of metabolites of arachidonic acid by P450s in neurite outgrowth of these cells.

## 2 MATERIALS AND METHODS

### 2.1 Materials

Dulbecco's modified Eagle's medium (DMEM), Dilauroyl‐ phosphatidylcholine, and RN‐1747 were purchased from Wako Pure Chemicals (Osaka, Japan). Fetal calf serum (FCS), penicillin‐ streptomycin solution, ketoconazole, and arachidonic acid sodium salt were purchased from Sigma Chemical (St. Louis, MO). 5,6‐, 8,9‐, 11,12‐, and 14,15‐EET, 5‐, 8‐, 9‐, 11‐, 12‐, 15‐, 16‐, 17‐, 18‐, 19‐, and 20‐HETE, and capsazepine were purchased from Cayman Chemical Co. (Ann Arbor, MI). Antibodies against CYP2C11, 2C23, 2E1, 4A2, sEH, NADPH‐cytochrome P450 reductase, and β‐actin were prepared as described previously.^16^, ^17^, ^18^ β‐NADPH was purchased from Oriental Yeast Co. LTD (Tokyo, Japan), horse serum was from Equitech‐Bio (Kerrville, TX), and nerve growth factor (NGF) was form Alomone Labs (Jerusalem, Israel). HC067047 and Fura‐2 AM were from Abcam plc (Cambridge, UK). Fluo‐4AM was from AAT Bioquest (Sunnyvale, CA).

### 2.2 Cell culture and PC12 cell neurite extension

Rat pheochromocytoma (PC12) cells were obtained from RIKEN cell bank (Tsukuba, Japan). Cells were cultured in DMEM containing 10% horse serum, 5% fetal calf serum(FCS), penicillin (100 units/mL), and streptomycin (100 μg/mL), and maintained at 37°C in 5% CO_2_ and 95% air. For the neurite outgrowth assay, cells were seeded at 1 × 10^4^ cells per well in 24‐well plates. After 1 day, culture medium was replaced with DMEM containing 0.25% HS, 0.13% FCS, and NGF (50 ng/mL) with EETs, DHETs or HETEs at the indicated concentrations. Differentiated cells with neurites were defined as those with neurite length greater than the cell body of individual cell, and ratio of differentiated cells to total number of cells was determined. Neurite length of differentiated cells was measured using Image J.

### 2.3 Isolation of rat hippocampal neuronal calls

Neuron‐rich cultures were prepared from hippocampus of Wistar rats (Clea Japan, Inc., Tokyo) at embryonic day 18 as described previously.^19^, ^20^ Cells were plated at 1 × 10^5^ cells per cloning ring on the poly‐ethylene‐imine coated dish with Neurobasal Medium (Thermo Fisher Scientific, MA) containing 2% B27 Supplement (Thermo Fisher Scientific), penicillin (100 units/mL) (Thermo Fisher Scientific), streptomycin (100 μg/mL) (Thermo Fisher Scientific), 5 μg/mL Insulin (Sigma Chemical), and 0.5 mmol L^−1^ l‐Glutamine.^21^, ^22^ After 24 hours, the cloning ring was removed, and 14,15‐EET (100 nmol L^−1^), 14,15‐DHET (100 nmol L^−1^), or TRPV4 agonist RN‐1747 (10 μmol L^−1^) were added to the cell medium. After 24 hours, the length of neurite diffused into the free space from cells was measured. For immunofluorescence analysis, the cells exposed to 14,15‐EET for 48 hours were fixed with 4% PFA, following BSA blocking for 1 hour, and incubated with anti‐neurofilament antibody (Cell Signaling), and Dylight594‐conjugated secondary antibody (Vector Laboratories, CA, USA) in Immuno‐enhancer solution (Wako, Osaka, Japan). Image was obtained by confocal microscopy (Nikon A1, Nikon, Tokyo, Japan). All experiments were conducted in accordance with guidelines on the welfare of experimental animals and with the approval of the Ethics Committee on the use of animals of Kwansei Gakuin University.

### 2.4 Arachidonic acid metabolism by P450s

Purification of rat P450 (CYP1A1, 1A2, 2A1, 2B1, 2C11, 2C13, 2C23, 2D1, 2E1, 2J3, 4A2, and 4F1), NADPH‐cytochrome P450 reductase, and cytochrome b_5_ was performed as described previously.^23^, ^24^ The reaction of arachidonic acid with P450 was carried out by the method described previously with a brief modification.^6^, ^23^, ^25^ As some of the arachidonic acid purchased from Sigma was converted to HETEs or EETs by autoxidation, the mixture was separated by HPLC and purified arachidonic acid was used for the substrate of P450. P450 (50 pmol) with cytochrome b_5_ (50 pmol), NADPH‐cytochrome P450 reductase (0.3 units), and dilauroylphosphatidylcholine (5 μg) was incubated with 100 μmol L^−1^ arachidonic acid and 1 mmol L^−1^ NADPH in 0.1 mol L^−1^ phosphate buffer at pH 7.4 containing 10 mmol L^−1^ MgCl_2_ and 150 mmol L^−1^ KCl for 15 minute at 37°C. One unit of the NADPH‐cytochrome P450 reductase activity was defined as the amount of reductase catalyzing the reduction of 1 μmol of cytochrome c per min. The final volume of the reaction mixture was 0.5 mL. The reaction was stopped by adding 100 μL of acetonitrile, and the metabolites were extracted with 2 mL of ethyl acetate. After drying the organic layer under nitrogen, the resulting residue was dissolved in ethanol and analyzed by UPLC/ESI/MS. Chromatography was performed with a C18 reversed phase column (TSK‐GEL ODS‐140HTP, 2.1 × 100 mm) and the UPLC system (ACQUITY UPLC system, Waters, Milford, MA). Mobile phase A (20% methanol and 0.1% acetic acid) and mobile phase B (80% acetonitrile, 20% methanol, and 0.1% acetic acid) were used, and chromatography was done at a flow rate of 0.2 mL/minute by gradient elution as follows: a linear gradient from 100% A to 60% B at 0‐18 minute, 60% B at 18‐23 minute a linear gradient from 60% B to 100% B at 23‐25 minute, and 100% B at 25‐26 minute. Mass spectrometry was carried out using a Nanofrontier LD mass spectrometer (Hitachi, Tokyo, Japan) and then ionized by electrospray ionization (ESI). ESI was accomplished in the negative ion mode with a spray potential of 3200 V. The analytes were detected by a tandem TOF monitored for total ions at *m*/*z* 319.2 for HETEs or EETs. The amount of produced HETEs and EETs was determined by a calibration curve prepared with authentic metabolites.

### 2.5 Calcium flux assay

PC12 cells were seeded in poly‐l‐lysine‐coated dishes. After incubation for 24 hours, cells were treated with 50 ng/mL NGF and cultured for 2 days. Cells were washed with PBS and incubated with 5 μg/mL Fura‐2 AM in Recording medium (20 mmol L^−1^ HEPES, 115 mmol L^−1^ NaCl, 5.4 mmol L^−1^ KCl, 0.8 mmol L^−1^ MgCl_2_, 1.8 mmol L^−1^ CaCl_2_, 13.8 mmol L^−1^ glucose, pH 7.4) for 1 hour at 37°C. After washing with PBS, Recording medium was added to the dishes. Cells were stimulated with EET or DHET, and the ratio of fluorescence intensity was monitored at 340/510 nm and 380/510 nm (excitation/emission) every 0.5 second for 1 minute by an EnVision 2104 Multilabel Reader (Perkin Elmer, Foster, CA). Rat neuronal cells were isolated and seeded on the poly‐l‐lysine‐coated dishes. After 3 days in culture, cells were incubated with 7.5 μg/mL Fluo‐4AM in cell culture medium for 1 hour at 37°C. After washing with PBS, Recording medium was added to the dishes. Cells were stimulated with 14,15‐EET and/or HC067047, and the fluorescence intensity was monitored at 485/535 nm (excitation/emission) every 0.5 second for 1 minute by an EnVision 2104 Multilabel Reader.

### 2.6 Statistical analysis

The differential significance of the results obtained was determined by One‐way ANOVA followed by a Bonferroni/Dunn post hoc test, and *P *<* *0.05 was considered statistically significant.

## 3 RESULTS

### 3.1 Effects of EETs and HETEs on NGF‐induced neurite outgrowth of PC12 cell

**Figure 1:**
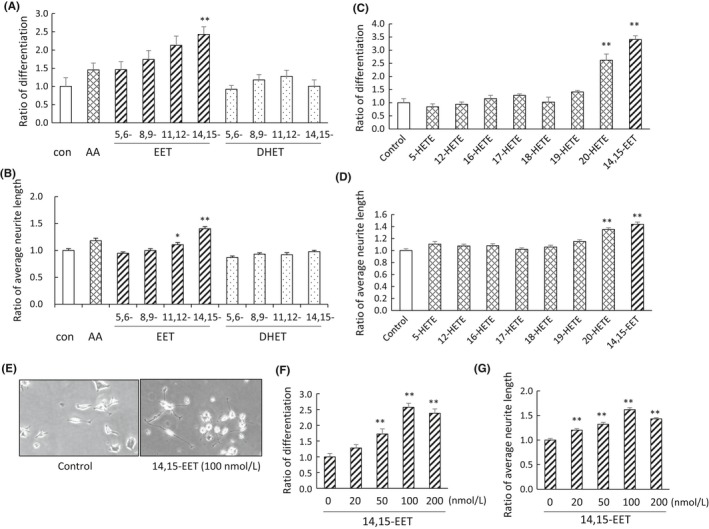
Enhancement of NGF‐induced PC12 cell neurite outgrowth by arachidonic acid metabolites. (A‐D) Arachidonic acid (AA), EETs, DHETs, or HETEs were added to cells at a concentration of 100 nmol L^−1^ in the presence of 50 ng/mL NGF for 48 hours. Number of differentiated cells with neurites those length was longer than the cell body was counted, and the ratio of differentiated cells to total number of cells was determined from four different dishes (A and C). Control value was set at 1.0. The average neurite length of cells was quantified from 100 (B) or 80 (D) cells. (E) Control value was set at 1.0. Representative image of PC12 cells treated with 100 nmol L^−1^ 14,15‐EETs in the presence of 50 ng/mL NGF for 48 hours. (F and G) Cells were treated with 14,15‐EET at a several concentrations with NGF for 48 hours. The ratio of differentiated cells to total cells (F), and the average neurite length of 100 cells (G) were quantified. Values are the mean ± SE. **P <* *0.01, *P <* *0.05 compared with control

As unsaturated fatty acids, such as arachidonic acid, are reported to enhance PC12 cell neurite outgrowth, effects of arachidonic acid metabolites, including EETs and HETEs, on neurite outgrowth were investigated. 100 nmol L^−1^ 14,15‐EET efficiently enhanced NGF‐induced cell differentiation by 240% and neurite extension by 140% compared with control, although effects of 100 nmol L^−1^ arachidonic acid on them were small (Figures 1A, B and F). DHETs, which are hydrolyzed products of EETs, affected neither cell differentiation nor neurite length (Figures 1A and B). The effects of 14,15‐EET were the greatest at 100 nmol L^−1^ during 20‐200 nmol L^−1^ (Figures 1F and G), and were not observed in the absence of NGF (data not shown). 20‐HETE also enhanced neurite outgrowth, but the other HETEs did not affect it (Figures 1C and D).

### 3.2 Quantification of HETEs and EETs produced by P450 isoforms

**Figure 2:**
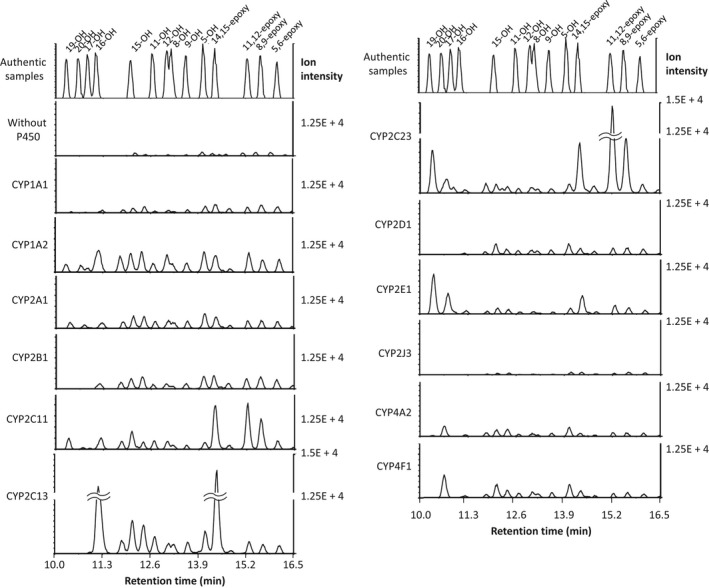
Production of EETs and HETEs by rat P450s. Fifty pmol of purified rat P450 with cytochrome b_5_, NADPH‐cytochrome P450 reductase, and dilauroylphosphatidylcholine was incubated with 100 μmol L^−1^ arachidonic acid and NADPH for 15 minutes, and the metabolites were analyzed with a UPLC‐mass spectrometer. The analytes were detected by a tandem TOF monitored by total ions at *m*/*z* 319.2

5‐, 8‐, 9‐, 11‐, 12‐, 15‐, 16‐, 17‐, 18‐, 19‐, and 20‐HETE, and 5,6‐, 8,9‐, 11,12‐, and 14,15‐EET were simultaneously separated by a mass spectrometer equipped with UPLC capable of high resolution. Using this LC‐MS system, HETEs and EETs produced by purified native CYP1A1, 1A2, 2A1, 2B1, 2C11, 2C13, 2C23, 2D1, 2E1, 2J3, 4A2, and 4F1 were quantitated (Figure 2, and Tables 1 and 2). CYP1A2, 2A1, 2B1, 2C11, 2C13, 2C23, 2E1, 4A2, and 4F1 efficiently produced HETEs or EETs. CYP1A2 produced 5‐, 9‐,11‐,12‐,15‐, 16‐, 18‐, and 19‐HETE, with particularly high amounts of 16‐HETE. CYP2A1, 2B1 and 2C13, and 2C23 produced 5‐, 8‐, 9‐, 11‐, 12‐, 15‐, and 16‐HETE, and CYP2C13 abundantly produced 16‐HETE. CYP2C23 and 2E1 efficiently produced 19‐HETE (ω‐1 hydroxylated arachidonic acid). CYP4A2 and 4F1 are fatty acid ω‐hydroxylases, but CYP4A2 produced 5‐ and 15‐ HETE as well as 20‐HETE (ω‐hydroxylated arachidonic acid). CYP4F1 produced several hydroxylated arachidonic acids as well as 20‐HETE. Regarding EETs, CYP1A1, 1A2, 2A1, 2B1, 2C11, 2C13, 2C23, and 2D1 produced four EETs, and CYP2C13 most efficiently produced 14, 15‐EET. 11,12‐EET was most efficiently produced by CYP2C23. Dihydroxyeicosatrienoic acids (DHETs) were not produced in these conditions.

**Table 1:** Hydroxylation activities of P450s toward arachidonic acid

P540 isoforms	pmol/min/nmol P450										
	5‐OH	8‐OH	9‐OH	11‐OH	12‐OH	15‐OH	16‐OH	17‐OH	18‐OH	19‐OH	20‐OH
CYP1A1	n.d.	n.d.	n.d.	n.d.	n.d.	n.d.	n.d.	n.d.	n.d.	n.d.	n.d.
CYP1A2	29.8	n.d.	14.1	14.2	41.6	25.4	53.8	n.d.	10.9	12.8	n.d.
CYP2A1	19.4	6.2	10.0	10.8	12.3	14.6	11.4	n.d.	5.6	9.3	n.d.
CYP2B1	14.6	n.d.	7.5	6.0	8.8	12.8	10.7	n.d.	n.d.	n.d.	n.d.
CYP2C11	5.3	5.7	n.d.	12.7	11.8	23.9	21.3	n.d.	n.d.	15.7	n.d.
CYP2C13	32.0	16.5	18.8	29.4	15.8	47.6	181.8	n.d.	n.d.	n.d.	n.d.
CYP2C23	11.5	5.7	6.2	6.6	10.8	9.1	6.3	8.5	n.d.	78.7	31.4
CYP2D1	12.0	5.3	6.1	5.7	7.0	13.6	n.d.	n.d.	n.d.	n.d.	n.d.
CYP2E1	n.d.	n.d.	n.d.	n.d.	n.d.	5.0	5.2	n.d.	42.0	72.0	n.d.
CYP2J3	n.d.	n.d.	n.d.	n.d.	n.d.	n.d.	n.d.	n.d.	n.d.	n.d.	n.d.
CYP4A2	9.6	n.d.	n.d.	n.d.	n.d.	6.9	n.d.	n.d.	n.d.	n.d.	18.9
CYP4F1	15.5	5.8	6.0	6.2	7.9	17.7	n.d.	n.d.	n.d.	n.d.	38.9

P450 (50 pmol) with cytochrome b_5_ (50 pmol), NADPH‐cytochrome P450 reductase (0.3 units), and dilauroylphosphatidylcholine (5 μg) was incubated with 100 μmol L^−1^ arachidonic acid and 1 mmol L^−1^ NADPH for 15 minutes at 37°C, and the metabolites were analyzed by LC‐MS. n.d. indicates activities of less than 5.0 pmol/min/nmol of P450.

**Table 2:** Epoxidation activities of P450s toward arachidonic acid

P540 isoforms	pmol/min/nmol P450			
	5,6‐epoxy	8,9‐epoxy	11,12‐epoxy	14,15‐epoxy
CYP1A1	2.3	4.0	1.9	5.1
CYP1A2	7.6	8.0	13.2	12.5
CYP2A1	2.6	3.6	3.3	9.1
CYP2B1	5.0	8.4	6.0	7.6
CYP2C11	3.9	23.5	35.4	35.7
CYP2C13	4.0	5.3	8.1	83.7
CYP2C23	5.5	51.9	91.4	44.3
CYP2D1	1.9	3.3	2.6	3.6
CYP2E1	n.d.	2.4	4.2	15.2
CYP2J3	n.d.	n.d.	n.d.	n.d.
CYP4A2	n.d.	n.d.	n.d.	1.1
CYP4F1	n.d.	1.2	n.d.	3.5

P450 (50 pmol) with cytochrome b_5_ (50 pmol), NADPH‐cytochrome P450 reductase (0.3 units), and dilauroylphosphatidylcholine (5 μg) was incubated with 100 μmol L^−1^ arachidonic acid and 1 mmol L^−1^ NADPH for 15 minutes at 37°C, and the metabolites were analyzed by LC‐MS. n.d. indicates activities of less than 1.0 pmol/min/nmol of P450.

### 3.3 Presence of P450s producing 14,15‐EET in PC12 cells

**Figure 3:**
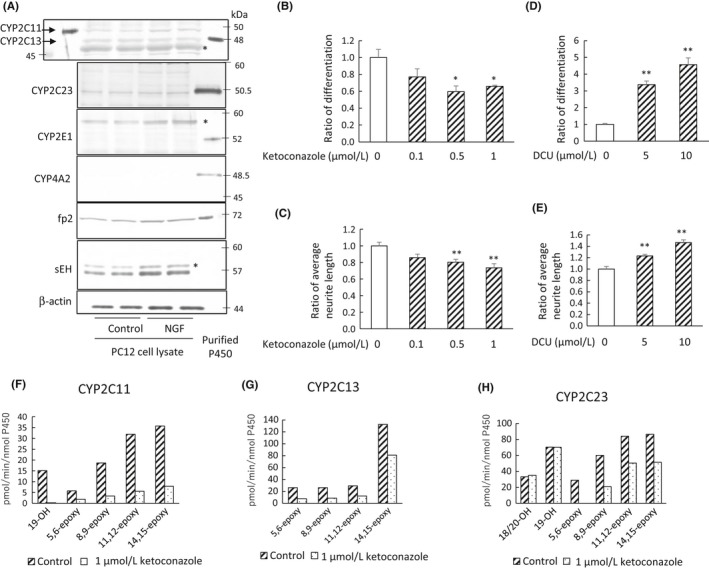
Inhibition of PC12 cell neurite outgrowth by a P450 inhibitor. (A) The protein expression of 14,15‐EET‐ producing P450s (CYP2C11, 2C13, 2C23, and 2E1), 20‐HETE‐ producing P450 (CYP4A2), NADPH‐cytochrome P450 reductase (fp2), and sEH in PC12 cells with or without 50 ng/mL NGF for 48 hours was detected by western blotting. The asterisks indicate nonspecific bands. The purified rat P450s for the arachidonic acid‐metabolizing assay were used as authentic controls. (B and C) Ketoconazole (0.1‐1 μmol L^−1^) was added to cells with 50 ng/mL NGF for 48 hours. Number of differentiated cells with neurites those length was longer than the cell body was counted, and the ratio of differentiated cells to total number of cells was determined from four different dishes (B). Control value was set at 1.0. The average neurite length of 80 cells were quantified (C). Control value was set at 1.0. (D and E) An inhibitor of sEH, N,N′‐dicyclohexylurea (DCU) was added to PC12 cells with 50 ng/mL NGF, and the ratio of differentiated cells to total cells (D) and the average neurite length of 100 cells (E) was measured after 48 hours. Values are the mean ± SE. **P < 0.01, *P < 0.05. (F‐H) CYP2C11, 2C13, or 2C23 was reacted with arachidonic acid in the presence (dotted bar) or absence (hatched bar) of 1 μmol L^−1^ ketoconazole, and the metabolites were analyzed by LC‐MS

We found that the most effective arachidonic acid metabolites to enhance neurite outgrowth of PC12 cells were 14,15‐EET which mainly produced by CYP2C and 2E1, and 20‐HETE produced by CYP4A (Figures 1 and 2). Next, we investigated protein levels of P450s which produce 14,15‐EET or 20‐HETE in PC12 cells (Figure 3A). CYP2C11, 2C13, and 2C23 were clearly detected in PC12 cells. However, CYP4A2, which produces 20‐HETE, was not detected. NADPH‐cytochrome P450 reductase and sEH proteins were detected in PC12 cells.

### 3.4 Inhibition of PC12 cell neurite outgrowth by P450 inhibitors

To investigate the contribution of 14,15‐EET‐producing P450 to PC12 cell neurite outgrowth, cells were treated with P450 inhibitors. First, we chose a general P450 inhibitor, SKF525A, but it was toxic at the working concentration. We used ketoconazole, which is an inhibitor for CYP3A P450s, but it can inhibit CYP2C P450s and EETs synthesis at much lower concentrations than SKF525A.^26^, ^27^ Ketoconazole (0.5 and 1 μmol L^−1^) inhibited neurite outgrowth of PC12 cells (Figures 3B and C), but did not affect cell viability. We confirmed that ketoconazole (1 μmol L^−1^) inhibited production of 14, 15‐EETs by CYP2C11, 2C13 and 2C23 from arachidonic acid (Figures 3F‐H). We also used MS‐PPOH, CYP epoxygenase inhibitor, however, inhibitory effects of 1 or 5 μmol L^−1^ MS‐PPOH on CYP2C11 epoxygenase activity and neurite outgrowth were lower than that of 1 μmol L^−1^ ketoconazole (data not shown). Pozzi et al also have shown that 1 μmol L^−1^ ketoconazole efficiently inhibited EET synthase, but not 1 μmol L^−1^ MS‐PPOH.^28^ On the other hand, the sEH inhibitor, DCU, enhanced NGF‐induced neurite outgrowth (Figures 3D and E).

### 3.5 Inhibition of calcium influx and neurite outgrowth in PC12 cells by TRP inhibitors

**Figure 4:**
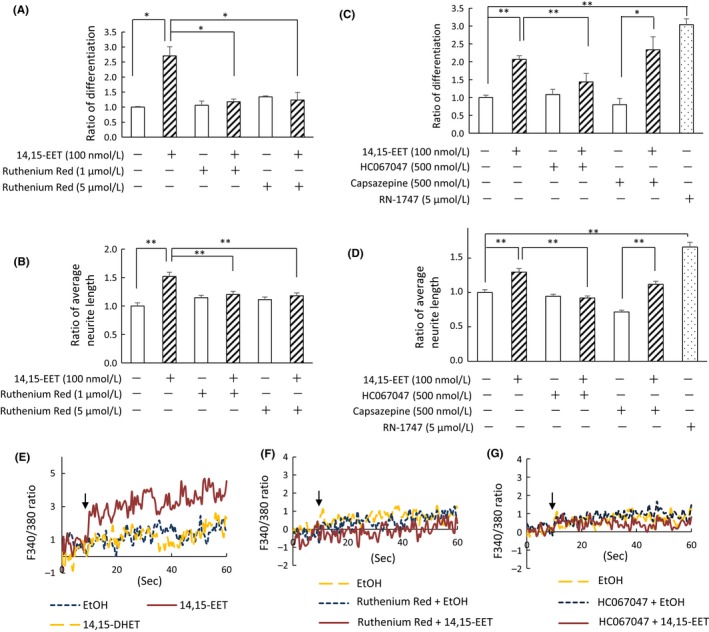
Inhibition of EET‐enhanced neurite outgrowth by TRPV4 inhibitor. (A‐D) The nonselective TRP inhibitor Ruthenium Red (1 or 5 μmol L^−1^), TRPV4 inhibitor HC067047 (500 nmol L^−1^), or TRPV1 inhibitor capsazepine (500 nmol L^−1^) was added to PC12 cells with 100 nmol L^−1^ 14,15‐EET and 50 ng/mL NGF for 48 hours. 5 μmol L^−1^ RN‐1747, TRPV4 agonist, was added to cells with NGF for 48 hours. Number of differentiated cells with neurites those length was longer than the cell body was counted, and the ratio of differentiated cells to total number of cells was determined from four different dishes (A and C). Control value was set at 1.0. The average neurite length of 80 (B) or 100 (D) cells were quantified. Control value was set at 1.0. Values are the mean ± SE. **P < 0.01, *P < 0.05 (E) Cells were cultured with 50 ng/mL NGF for 48 hours, and changes in the concentration of cellular calcium ion by the addition of ethanol (dotted line), 170 nmol L^−1^ 14,15‐EET (solid line), or 14,15‐DHET (dashed line) were detected by Fura‐2AM. The arrow indicates the time that EET or DHET was added. (F and G) The concentration of calcium ion was measured by adding ethanol (dotted line) or 170 nmol L^−1^ 14,15‐EET (solid line) with 500 nmol L^−1^ Ruthenium Red (D) or 500 nmol L^−1^ HC067047 (E). The arrow indicates the time that EET and the inhibitor were added

Although the receptors for EETs remain unknown, EETs has been recently shown to activate TRPV1 and TRPV4.^13^, ^29^ These TRP proteins were expressed in PC12 cells.^30^, ^31^ We investigated the effects of TRP inhibitors on neurite outgrowth and calcium ion influx in PC12 cells to speculate the mechanism of how 14,15‐EET regulates PC12 cell neurite outgrowth. A broad inhibitor of TRP, ruthenium red, inhibited both the cell differentiation and neurite extension enhanced by 14,15‐EET (Figures 4A and B). Furthermore, specific inhibitor of TRPV4, HC067047 (500 nmol L^−1^), but not the TRPV1 inhibitor capsazepine (500 nmol L^−1^), inhibited the neurite outgrowth enhanced by 100 nmol L^−1^ 14,15‐EET (Figures 4C and D). Everaerts et al. have showed that HC‐067047 inhibited 4α‐phorbol 12,13‐didecanoate‐stimulated rat TRPV4 activity with IC50 values of 133 ± 25 nmol L^−1^, and that HC067047 is specific for TRPV4, because the IC50 value for TRPV4 were 100‐fold higher for the closely related channels TRPV1, TRPV2, and TRPV3, and 10‐fold higher for TRPM8 and hERG.^32^ Furthermore, we used RN‐1747 which has been identified as a specific TRPV4 agonist with EC50 values of 4 μmol L^−1^ (rat TRPV4). 5 μmol L^−1^ RN‐1747 efficiently enhanced NGF‐induced cell differentiation and neurite extension (Figures 4C and D). We found that 14,15‐EET, but not 14,15‐DHET, increased the cytosolic calcium ion concentration of PC12 cells (Figure 4E), and this increase was inhibited by both ruthenium red and HC067047 (Figures 4F and G), suggesting that 14,15‐EET enhances neurite outgrowth through calcium influx, and activation of TRPV4 channel may be involved in this effects.

### 3.6 Enhancement of neurite outgrowth of rat primary cultured neuron by 14,15‐EET

**Figure 5:**
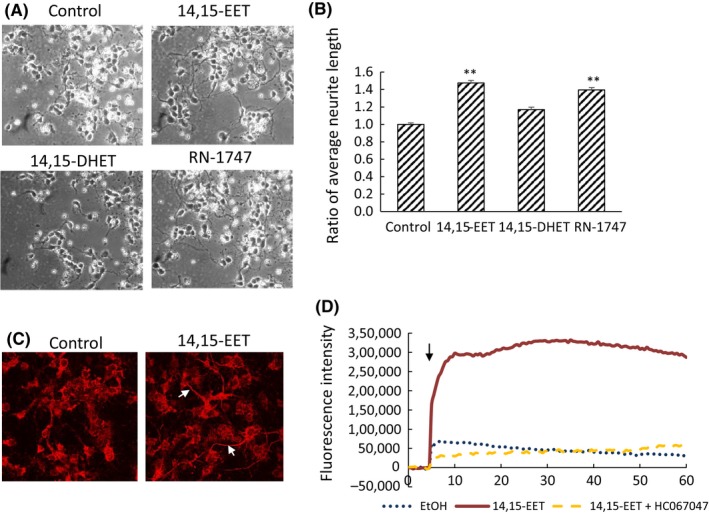
Enhancement of rat primary cultured neuronal cell outgrowth by 14,15‐EET. (A‐C) Rat neuronal cells were isolated from hippocampus of Wistar rats at embryonic day 18, and plated within a cloning ring on the poly‐ethylene‐imine coated dish. After 24 hours, the cloning ring was removed, and 14,15‐EET (100 nmol L^−1^), 14,15‐DHET (100 nmol L^−1^), or TRPV4 agonist RN‐1747 (10 μmol L^−1^) were added to the cell medium. After 24 hours, the length of neurite diffused into the free space from 200 cells was measured (A and B). Values are the mean ± SE. The cells exposed to 14,15‐EET for 48 hours, were fixed with 4% PFA, and analyzed by immunofluorescence with anti‐neurofilament antibody (C). (D) Isolated rat neuronal cells were cultured for 3 days, and incubated with Fluo‐4AM, and changes in the concentration of cellular calcium ion by addition of ethanol (dotted line), 200 nmol L^−1^ 14,15‐EET (solid line), or 200 nmol L^−1^ 14,15‐EET with 500 nmol L^−1^ HC067047 (dashed line). The arrow indicates the time that these reagents were added. **P < 0.01 compared with control

To investigate the effects of 14,15‐EET on neuronal cells under normal physiological condition, we isolated neuronal cells by rat fetal hippocampus. After 24 hours, 100 nmol L^−1^ 14,15‐EET, 14,15‐DHET, or 10 μmol L^−1^ RN‐1747 (TRPV4 agonist) was added, and cultured for additional 24 hours. As a result, 14,15‐EET enhanced neurite length of cells by 150% compared with control, but 14,15‐DHET did not (Figures 5A and B). In addition, 14,15‐EET increased neurofilament‐positive neurons which are thought to be mature axons^33^ (Figure 5C). In rat hippocampus, and cultured hippocampal neurons, expression of TRPV4 has been shown.^34^ We found that a TRPV4 agonist, RN‐1747 also enhanced neurite outgrowth of cells (Figure 5B). Furthermore, 14,15‐EET stimulated calcium influx of primary neuron‐rich cultures, and this effect was suppressed in the presence of TRPV4 inhibitor, HC067047. These results suggest that 14,15‐EET promoted neurite outgrowth of hippocampal neurons, and calcium influx by activation of TRPV4 may be involved in this effect.

## 4 DISCUSSION

Arachidonic acid, which is abundant in the brain has been shown to be involved in neurite outgrowth, and essential for infant growth and development. It has been reported that 200 μmol L^−1^ or 60 μmol L^−1^ arachidonic acid enhanced NGF‐induced neurite outgrowth of PC12 cells^35^, ^36^. We found that 100 nmol L^−1^ 14,15‐EET efficiently enhanced neurite outgrowth of PC12 cells and rat hippocampal neuronal cells. 100 nmol L^−1^ (=32.5 ng/mL) 14,15‐EET would be a physiological concentration because total hypothalamic EET concentration was estimated to be 120 ng/g wet tissue in rat.^37^

14,15‐EET was mainly produced by rat CYP2C11, 2C13, and 2C23 from arachidonic acid. CYP2C13 reveals relatively low activity toward drugs and testosterone.^38^, ^39^ We found that CYP2C13 efficiently produced 16‐HETE and 14,15‐EET from arachidonic acid. These results are consistent with those of El‐Sheberi and El‐Kadi^40^ using the microsomes of cells expressing recombinant rat CYP2C13. Although human CYP2J was isolated as an arachidonic acid epoxygenase,^41^ rat CYP2J3 exhibited little epoxidase activity toward arachidonic acid in this study.

In this study, we detected 14,15‐EET‐ producing P450s (CYP2C11, 2C13, and 2C23) in PC12 cells, and their inhibitor, ketoconazole, abrogated NGF‐enhanced neurite outgrowth, suggesting the contribution of these P450s to neurogenesis. Ketoconazole also inhibit EET‐induced relaxation in monkey cerebral artery.^42^ Expression of CYP2C in rat brain has been shown in several reports.^23^, ^43^ CYP2C11 is expressed in perivascular nerves in rat brain,^44^ and CYP2C9 and 2C19 are detected in neuronal cells in humans,^45^ suggesting production of EETs in neuronal cells. In addition, release of EETs form astrocyte and cerebral vascular endothelium has been shown by many reports,^46^, ^47^, ^48^ suggesting the released EETs can activate outgrowth of neuronal cells. Astrocyte‐derived 14,15‐EET has been shown to be a protective effect on oxidative stress‐induced cell injury in dopaminergic neuronal cell.^49^

TRPV4 is a nonselective cationic channel known to be activated by several endogenous or synthetic chemicals.^50^ EETs and anandamide are endogenous activators of TRPV4, and EETs were reported to act through the TRPV4‐TRPC1‐Kca1.1 complex in smooth muscle cells as a vasodilator.^51^ TRPV4 has a putative EET‐recognition site in its cytoplasmic N‐terminal region,^52^ but further studies are necessary to elucidate the mechanism behind the activation of TRPV4 by 14,15‐EET inside or outside of the plasma membrane. In the brain, TRPV4 is expressed in the hippocampus and circumventricular organs, and is essential for axonal growth in early neuronal development as well as for neuron maintenance in adults.^53^, ^54^ PC12 cells express TRPV4, and knockdown of TRPV4 decreases PC12 cells neurite outgrowth.^30^ Consistent with this, we found that a broad inhibitor of TRP, ruthenium red, and a specific inhibitor of TRPV4 but not TRPV1, HC067047, inhibited the neurite outgrowth enhanced by 14,15‐EET. Furthermore, Increase in cytosolic calcium ion concentration by 14,15‐EET was involved in TRPV4. Increased cellular calcium ion concentrations have been shown to induce NGF‐induced neurogenesis via promotion of actin polymerization.^30^ In addition to TRPV4, 14,15‐EET has been shown to activate several ion channels including calcium‐activated potassium channels and voltage‐gated calcium channels. Therefore, it is not excluded the possibility that 14,15‐EET can work on these channels, not only TRPV4 for stimulation of calcium influx. Furthermore, it has been shown that TRPV4 channels are functionally coupled with low‐conductance calcium‐activated potassium channels in paraventricular nucleus neurons.^55^ Therefore, it is possible that calcium ion entry by 14,15‐EET‐elicited activation of TRPV4 is amplified by subsequent activation of other channels.

Our study suggests that arachidonic acid metabolites, 14,15‐EETs, produced by cytochrome P450 contribute to neurite outgrowth through activation of TRPV4. Regulation of EET levels using sEH inhibitors, which hydrolyze EETs in the brain, has been explored as therapy for cerebral vascular diseases, such as stroke, because EETs will improve cerebral blood flow as vasodilators.^14^ Therefore, the increase in EET levels by suppression of sEH activity in neuronal cells may also be effective in enhancing neurogenesis.

## AUTHOR CONTRIBUTIONS

Participated in research design: Oguro A. and Imaoka S.

Conducted experiments: Oguro A.

Conducted experiments for isolation of rat hippocampal neuronal cells: Kudoh NS.

Isolation of rat hippocampal neuronal cells: Inoue T.

Performed data analysis: Oguro A.

Wrote or contributed to the writing of the manuscript: Oguro A. and Imaoka S.

## DISCLOSURES

None declared.
